# Analysis of risky sexual behaviors among male college students who were sexually active in Sichuan, China: a cross-sectional survey

**DOI:** 10.1186/s12981-024-00636-1

**Published:** 2024-07-26

**Authors:** Yingxue Dai, Yajie Li, Dinglun Zhou, Jianxin Zhang

**Affiliations:** 1 Chengdu Center for Disease Control and Prevention, Chengdu, China; 2 West China School of Public Health and West China Fourth Hospital, Sichuan University, Chengdu, China; 3 Center for Disease Control and Prevention of Tibet autonomous region, Lhasa, China

**Keywords:** HIV, Cognition, Risky sexual behaviors, Male, College students

## Abstract

**Background:**

Males have accounted for a significant share of new HIV infections among young people in the recent years. This study aimed to identify the factors associated with risky sexual behaviors, including early sexual debut, multiple sexual partnership and condomless sex, among sexually active male college students and provide implications for tailored health interventions.

**Methods:**

The cross-sectional study was conducted from December 2020 to December 2021 in 16 colleges that were located in Sichuan Province, one of the high-risk areas in China. Overall 1640 male college students who reported sexually experienced were analyzed in this study. Multivariable logistic regression analysis was applied to determine factors associated with early sexual debut, multiple sexual partnership and condomless sex.

**Results:**

The average age of included male students was 19.95 ± 1.56. Of them, 27.74% initiated sexual behavior early, 48.60% reported multiple sexual partnership, and 16.52% did not use condoms at the latest sexual intercourse. Students who were younger (age ≤ 19, AOR = 7.60, 95%CI: 4.84–11.93; age20-21, AOR = 3.26, 95%CI: 2.04–5.21) and self-identified as sexual minorities (AOR = 2.38, 95%CI: 1.69–3.36) were more likely to have early sexual debut. The odds of having multiple sexual partners were higher among those who were ethnic minorities (AOR = 1.79, 95%CI: 1.33–2.41) and accepted extramarital sex (AOR = 1.33, 95%CI: 1.03–1.71). The likelihood of engaging in condomless sex at the latest sexual intercourse was lower among those who had sufficient knowledgeable about HIV (AOR = 0.63, 95%CI: 0.44–0.89), were very confident in condom use efficacy (AOR = 0.26, 95%CI: 0.16–0.43) and confident (AOR = 0.48, 95%CI: 0.34–0.69). Early sexual debut was positively associated with multiple sexual partnership (AOR = 3.64, 95%CI: 2.82–4.71) and condomless sex at the latest intercourse (AOR = 1.53, 95%CI: 1.07–2.20), respectively.

**Conclusion:**

Early sexual debut, multiple sexual partnership and condomless sex were of considerable concern among male college students. Comprehensive sex education curricula were advised by developing customized information on HIV prevention, sexuality and empowering students with assertiveness and negotiation skills with regard to condom use during and before college.

## Introduction

HIV infection among young people continued to be a pervasive public health issue globally [1–3]. In 2021, approximately 1.5 million people were newly infected with HIV worldwide, and nearly one third of them were young people between the ages of 10 to 24 [1]. In China, although the national-level prevalence was comparatively low [4], the transferred newly affected persons among young people experienced an alarming surge in recent years [5, 6]. According to a countrywide surveillance report, new infections in age group 15–24 years had a 3.3-fold increase from 2010 to 2019, reaching over 3400 cases per year [7].

Males dominated the new HIV infections among young people in China, constituting more than 95% in each of the recent 10 years [7]. This gender distribution disparity was similar to that in Western and Central Europe and North America but more discernible in China [2]. The leading transmission route for Chinese young males was sexual contact, with homosexual transmission accounting for more than 80% and heterosexual transmission representing about 16% [7, 8].

Sexual activity during college life was common for male students worldwide [9–11]. A study including 25,553 students from 55 universities in the U.S reported that the proportion of male college students having sexual behavior has exceeded more than 90% [10]. In China, along with sexual cultural integration and changes in sexual attitude [12], this proportion has witnessed a pronounced increase in recent years. A meta-analysis indicated the percentage of sexually experienced male students was 13.7% in the early 21st century [13], but dramatically ascended up to 52.1% in 2019 and progressively grew by ages [14, 15].

Along with sexual exploration, male college students may engage in risky sexual behaviors like early sexual debut, multiple sexual partner and condomless sex [14, 16–19]. In China, 37.3% of male sexually active students had multiple sexual partners during the previous 6 months [14], and the proportions of consistent condom use with casual partners were only 46.9% for hetero-sex and 36.4% for homo-sex [19]. The impact of risky sexual behaviors among males was enormous, such as increasing the risks of contracting Sexually transmitted diseases (STDs) [20] and unwanted pregnancy in their female partners [21], which further affected their academic career and life plan. Nine consecutive cross-sectional surveys showed that men who have sex with men who never or sometimes used condoms were 1.561 times more likely to get recently infected with HIV, and the odds of being infected with HIV was 2.141 for those who had multiple sexual partners [22].

Several studies have reported sexual knowledge, attitude and behaviors among this vulnerable age group [14, 17, 23–26]. For example, inconsistent condom use was indicated associated with grades, sexual orientation, monthly expenditure [14], HIV knowledge [25] and condom efficacy [23]. To monitor the evolution changes of HIV epidemic, more evidence in identifying factors of leverage for effective interventions was still needed, especially among male college students, the group with a particularly high risk of HIV infection.

Sichuan province has more than 100,000 people living with HIV/AIDS in 2019 [27], taking up about 10% in China [28]. Also, it has the largest number of new infections among young people from 2010 to 2019 across the country [7]. However, there is little knowledge about epidemiological characteristics and associations of sexual behaviors among young people in Sichuan. To address this gap, this study aimed to (1) understand the prevalence and associated factors of risky sexual behaviors among male college students in Sichuan, and; (2) explore practical preventing approaches for risky sexual behaviors for college students.

## Materials and methods

### Participants and procedure

This was a cross-sectional survey conducted from December 2020 to December 2021 in Sichuan province. Sixteen vocational colleges, out of 79, were selected as the study sites. The selected colleges were distributed in 6 cities, representing different levels of socioeconomic status in Sichuan. A specific QR code, which linked to a self-administered online anonymous questionnaire, was designed and attributed to trained teachers from the 16 vocational colleges. The teachers introduced the background of the survey and QR code to potential participants by online chat tools and classes. Students who were interested in the survey could scan the QR code with their phones and were required to fulfill the questionnaire within 20 min.

### Inclusion criteria

Participants were included in this study if they satisfied the following criteria: (i) males, (ii) aging 18 and above, (iii) having had oral-genital, vaginal, or anal intercourse. Totally, 13,945 students completed the survey, of which 6750 were males. Among them, 1640 students met the inclusion criteria of this study, accounting for 24.30% of the total number of males.

### Measures

This study measured sociodemographic characteristics of the participants, including age, hometown, ethnic group, sexual orientation, etc. Sexual orientation was the enduring pattern of an individual’s sexual attraction. Further, participants’ awareness of HIV/AIDS-related knowledge, sexual attitudes, condom use efficacy, perceived risk of HIV infection and risky sexual behaviors were explored. The outcome of interest in the study was risky sexual behaviors, including early sexual debut, multiple sexual partners and condomless sex at the latest sexual intercourse.

### Operational definitions

In this study, sexual orientation was defined by asking the participants, “what do you think your sexual orientation is”. Four answers were listed, heterosexual, homosexual, bisexual and unclear. We further grouped the answers into two categories, heterosexual and sexual minorities which included homosexual, bisexual and unclear [29].

Awareness of HIV/AIDS-related knowledge was measured by a number of 8 items, which was designed by National Center for AIDS/STD Control and Prevention, China CDC [30], and widely used in national surveillance [14, 31, 32]. The answers to each item included three options (true, false and unclear). Each correct answer was given one score. If the total scores reach 6 or above, it implied that the participant had a good knowledge of HIV prevention [32].

Sexual attitudes were explained by attitudes towards premarital sex, unmarried cohabitation and extramarital sex. There were five response options listing from “totally disagree” to “totally agree”. In this analysis, options were dichotomized into agreement (totally agree/agree) and disagreement (totally disagree/disagree/do not care).

Condom use efficacy, conceptualized as a person’s confidence to use condoms [33], was evaluated by three questions adapted from the Condom-Use Efficacy Scale (CUSES) [21, 34]. The questions, aiming to understand the ability to insist, negotiate and reject concerning using condoms, were listed as follows, “I feel confident in using condoms when having sexual intercourse”, “I feel confident in negotiating with sexual partners about condom use before sex”, “I feel confident in rejecting if my sexual partner did not want to use condoms”. Five options were provided, from 1 point (totally disagree) to 5 points (totally agree). The measurement scores were categorized into three groups, “unconfident, confident and very confident “, based on 3–9, 10–12, 13–15 points respectively. The Cronbach alpha coefficient was 0.772. Perceived risk of HIV infection was evaluated by asking, “do you think you are at risk of getting infected with HIV”, with two answers provided, “yes” and “no”.

In this study, risky sexual behaviors consisted of early sexual debut, having multiple sexual partners, and condom use at the latest intercourse. Early sexual debut was defined as having sex before the age of 18 [35, 36]. Having multiple sexual partners was identified when having more than one sexual partner in the previous 12 months before the survey [37].

### Statistical analysis

Data were recorded by Wenjuanxing, a widely used online survey tool in China, and imported into SPSS 17 for Windows (SPSS Inc., Chicago, Illinois, USA) for further statistical analysis. Categorical variables were presented in terms of frequencies and proportions, and continuous variables were demonstrated with mean and standard deviation (SD). Chi-square was applied to compare categorical variables. Univariate logistic regression analysis was used in the bivariate analysis to document variables associated with early sexual debut, multiple sexual partnership, and condom use at the latest sexual intercourse. Since the knowledge about HIV, sexual attitudes, and condom use efficacy, etc. might vary a lot along with time span, sexual experience, and received health education [38, 39], only sociodemographic characteristics were included into the analysis of influential factors with early sexual debut. Variables significant at p-value < 0.10 and sexual orientation regardless of p-value were included in the Multivariable logistic regression model to obtain adjusted odds ratios (AOR) and 95% confidence intervals (CI). The significance level was set at *p* < 0.05. There was no evidence of multicollinearity among the variables included in the Multivariable regression models.

## Results

### Characteristic of the participants

Among the 1640 participants, the mean age was 19.95 years (SD = 1.56). Most of the male students resided in Sichuan province (81.52%) and were Han ethnic (80.98%). Slightly more than half (53.84%) lived in towns or cities. Parents’ education levels were mainly junior high school level or below. About one-tenth (10.98%) identified themselves as sexual minorities, with 3.23% as homosexual and 2.62% as bisexual (Table 1).

**Table 1:** Characteristics and risky behaviors of the participants, *n*(%)

		Characteristics	early sexual debut	Multiple sexual partnership	Condomless sex
Age(year)	≤ 19	781(47.62)	310(39.69)	369(47.25)	142(18.18)
	20–21	560(34.15)	120(21.43)	290(51.79)	100(17.86)
	≥ 22	299(18.23)	25(8.36)	138(46.15)	29(9.70)
Household registration	Sichuan province	1337(81.52)	387(28.95)	651(48.69)	233(17.43)
	Other provinces	303(18.48)	68(22.44)	146(48.18)	38(12.54)
Hometown	Rural	757(46.16)	193(25.5)	353(46.63)	138(18.23)
	Town/city	883(53.84)	262(29.67)	444(50.28)	133(15.06)
Ethnic group	Han (majority ethnic group)	1328(80.98)	393(29.59)	624(46.99)	211(15.89)
	Ethnic minorities	312(19.02)	62(19.87)	173(55.45)	60(19.23)
Father educational background	Junior high school and below	1111(67.74)	288(25.92)	528(47.52)	196(17.64)
	Senior high school	331(20.18)	110(33.23)	169(51.06)	44(13.29)
	College and above	198(12.07)	57(28.79)	100(50.51)	31(15.66)
Mother educational background	Junior high school and below	1225(74.70)	335(27.35)	575(46.94)	225(18.37)
	Senior high school	286(17.44)	87(30.42)	162(56.64)	31(10.84)
	College and above	129(7.87)	33(25.58)	60(46.51)	15(11.63)
Monthly living expenses	Less than 1,000 RMB	710(43.29)	193(27.18)	335(47.18)	134(18.87)
	1,000-1999 RMB	756(46.10)	205(27.12)	357(47.22)	112(14.81)
	2000RMB and above	174(10.61)	57(32.76)	105(60.34)	25(14.37)
Family structure	Family with biological parents	1355(82.62)	372(27.45)	657(48.49)	219(16.16)
	Other family structures	285(17.38)	83(29.12)	140(49.12)	52(18.25)
Sexual orientation	Heterosexual	1460(89.02)	381(26.10)	688(47.12)	234(16.03)
	Sexual minorities	180(10.98)	74(41.11)	109(60.56)	37(20.56)
HIV knowledge	Insufficient	477(29.09)	-	548(47.12)	114(23.90)
	Sufficient	1163(70.91)	-	249(52.20)	157(13.50)
Attitude towards unmarried cohabitation	Disagreement	270(16.46)	-	126(46.67)	35(12.96)
	Agreement	1370(83.54)	-	671(48.98)	236(17.23)
Attitude towards premarital sex	Disagreement	244(14.88)	-	99(40.57)	28(11.48)
	Agreement	1396(85.12)	-	698(50.00)	243(17.41)
Attitude towards extramarital sex	Disagreement	1179(71.89)	-	539(45.72)	167(14.16)
	Agreement	461(28.11)	-	258(55.97)	104(22.56)
Condom use efficacy	Unconfident	449(27.38)	-	249(55.46)	153(34.08)
	Confident	682(41.59)	-	310(45.45)	88(12.9)
	Very confident	509(31.04)	-	238(46.76)	30(5.89)
Perceived risk of HIV infection	No	1424(86.83)	-	681(47.82)	216(15.17)
	Yes	216(13.17)	-	116(53.7)	55(25.46)
HIV test history	Yes	314(19.15)	-	184(58.6)	55(17.52)
	No	1326(80.85)	-	613(46.23)	216(16.29)
Experience of being in love	Once or less	449(27.38)	-	122(27.17)	73(16.26)
	Two times or more	1191(72.62)	-	675(56.68)	198(16.62)
Early sexual debut	No	1185(72.26)	-	464(39.16)	149(12.57)
	Yes	455(27.74)	-	333(73.19)	122(26.81)
Condom use at the first intercourse	No	485(29.57)	-	290(59.79)	213(43.92)
	Yes	1155(70.43)	-	507(43.90)	58(5.02)
Multiple sexual partnership	No	843(51.4)	-	-	108(12.81)
	Yes	797(48.60)	-	-	163(20.45)
Condom use at the latest intercourse	Yes	1369(83.48)	-	634(46.31)	-
	No	271(16.52)	-	163(60.15)	-

### Correlates with early sexual debut

Of the participants, the mean age at sexual debut was 17.98 years (SD = 1.85), and 455(27.74%) males initiated sexual behavior before 18 (Table 1). Univariate analysis indicated the following variables were associated with early sexual debut: age, household registration, ethnic group, father educational background and sexual orientation. By including the factors into Multivariable analysis, we found that a higher likelihood of early sexual debut was linked to aging 19 and below (AOR = 7.60, 95%CI: 4.84–11.93), aging 20–21(AOR = 3.26, 95%CI: 2.04–5.21), and being self-identified as sexual minorities (AOR = 2.38, 95%CI: 1.69–3.36) (Table 2).

**Table 2:** Factors correlated with early sexual debut

		Univariate analysis		Multivariable analysis	
		OR(95% CI)	*P* value	AOR(95% CI)	*P* value
Age(year)	≤ 19	7.21(4.67–11.13)	< 0.001	7.60(4.84–11.93)	< 0.001
	20–21	2.99(1.89–4.72)	< 0.001	3.26(2.04–5.21)	< 0.001
	≥ 22	1		1	
Household registration	Sichuan province	1			
	Other provinces	0.71(0.53–0.95)	0.023		
Hometown	Rural	1			
	Town/city	1.23(0.99–1.53)	0.060		
Ethnic group	Han (majority ethnic group)	1			
	Ethnic minorities	0.59(0.44–0.8)	0.001		
Father educational background	Junior high school and below	1			
	Senior high school	1.42(1.09–1.85)	0.009		
	College and above	1.16(0.83–1.62)	0.399		
Sexual orientation	Heterosexual	1		1	
	Sexual minorities	1.98(1.44–2.72)	< 0.001	2.38(1.69–3.36)	< 0.001

### Correlates with multiple sexual partners

The proportion of reporting multiple sexual partners was 48.60% (Table 1). According to the univariate analysis, ethic group, mother educational background, monthly living expenses, etc. were associated with multiple sexual partnership. By applying Multivariable analysis, participants who were ethnic minorities (AOR = 1.79, 95%CI: 1.33–2.41), had a senior high school educated mother (AOR = 1.57, 95%CI: 1.16–2.12), had more than 2000RMB as monthly living expenses (AOR = 1.55, 95%CI: 1.03–2.32), agreed with extramarital sex (AOR = 1.33, 95%CI: 1.03–1.71), had two times or more experiences of getting in love (AOR = 3.80, 95%CI: 2.92–4.94), and had early sexual debut (AOR = 3.64, 95%CI: 2.82–4.71) were more likely to have multiple sexual partnership. Those who used condoms at the first sex (AOR = 0.72, 95%CI: 0.54–0.95), and never tested HIV (AOR = 0.60, 95%CI: 0.45–0.81) had lower possibility of reporting multiple sexual partners (Table 3).

**Table 3:** Factors correlated with multiple sexual partnership

		Univariate analysis		Multivariable analysis	
		OR(95% CI)	*P* value	AOR(95% CI)	*P* value
Ethnic group	Han (majority ethnic group)	1			
	Ethnic minorities	1.4(1.1–1.8)	0.007	1.79(1.34–2.41)	< 0.001
Mother educational background	Junior high school and below	1			
	Senior high school	1.48(1.14–1.91)	0.003	1.57(1.16–2.12)	0.003
	College and above	0.98(0.68–1.41)	0.926		
Monthly living expenses	Less than 1,000 RMB	1			
	1,000-1999 RMB	1(0.82–1.23)	0.988		
	2000RMB and above	1.7(1.22–2.39)	0.002	1.55(1.03–2.32)	0.034
Sexual orientation	Heterosexual	1			
	Sexual minorities	1.72(1.26–2.36)	0.001		
Attitude towards premarital sex	Disagreement	1			
	Agreement	1.47(1.11–1.93)	0.007		
Attitude towards extramarital sex	Disagreement	1			
	Agreement	1.51(1.22–1.87)	< 0.001	1.33(1.03–1.71)	0.029
Condom use efficacy	Unconfident	1			
	Confident	0.67(0.53–0.85)	0.001		
	Very confident	0.71(0.55–0.91)	0.007		
HIV test history	Yes	1			
	No	0.61(0.47–0.78)	< 0.001	0.60(0.45–0.81)	0.001
Experience of being in love	Once or less	1			
	Two times or more	3.51(2.77–4.45)	< 0.001	3.80(2.92–4.94)	< 0.001
Early sexual debut	No	1			
	Yes	4.24(3.34–5.38)	< 0.001	3.64(2.82–4.71)	< 0.001
Condom use at the first intercourse	No	1			
	Yes	0.53(0.42–0.65)	< 0.001	0.72(0.54–0.95)	0.019
Condom use at the latest intercourse	Yes	1			
	No	1.75(1.34–2.28)	< 0.001		

### Correlates with condomless sex at the latest sexual intercourse

Overall, 271 (16.52%) did not use condoms at the latest sexual intercourse (Table 1). In the univariate analysis, influential factors included age, mother educational background, HIV knowledge, condom use efficacy, etc. From the Multivariable analysis, the likelihood of engaging in condomless sex was lower among those who had a senior high school educated mother (AOR = 0.49, 95%CI: 0.29–0.82), had sufficient knowledgeable about HIV (AOR = 0.63, 95%CI: 0.44–0.89), used condoms at the first intercourse (AOR = 0.08, 95%CI: 0.06–0.12), and being confident (AOR = 0.48, 95%CI: 0.34–0.69) and very confident (AOR = 0.26, 95%CI: 0.16–0.43) in condom use. The odds of having condomless sex increased among participants aging 19 and below (AOR = 1.91, 95%CI: 1.13–3.23), reporting perceived risk of HIV infection (AOR = 2.05, 95%CI: 1.33–3.16), and having early sexual debut (AOR = 1.53, 95%CI: 1.07–2.20) (Table 4).

**Table 4:** Factors correlated with condomless sex at the latest sexual intercourse

		Univariate analysis		Multivariable analysis	
		OR(95% CI)	*P* value	AOR(95% CI)	*P* value
Age(year)	≤ 19	2.07(1.35–3.16)	0.001	1.91(1.13–3.23)	0.016
	20–21	2.02(1.3–3.14)	0.002		
	≥ 22	1			
Household registration	Sichuan province	1			
	Other provinces	0.68(0.47–0.98)	0.040		
Hometown	Rural	1			
	Town/city	0.8(0.61–1.03)	0.085		
Father educational background	Junior high school and below	1			
	Senior high school	0.72(0.5–1.02)	0.063		
	College and above	0.87(0.57–1.31)	0.497		
Mother educational background	Junior high school and below	1			
	Senior high school	0.54(0.36–0.81)	0.003	0.49(0.29–0.82)	0.006
	College and above	0.58(0.33–1.02)	0.059		
Monthly living expenses	Less than 1,000 RMB	1			
	1,000-1999 RMB	0.75(0.57–0.98)	0.038		
	2000RMB and above	0.72(0.45–1.15)	0.167		
Sexual orientation	Heterosexual	1			
	Sexual minorities	1.36(0.92-2)	0.124		
HIV knowledge	Insufficient	1			
	Sufficient	0.50(0.38–0.65)	< 0.001	0.63(0.44–0.89)	0.008
Attitude towards unmarried cohabitation	Disagreement	1			
	Agreement	1.4(0.95–2.05)	0.086		
Attitude towards premarital sex	Disagreement	1			
	Agreement	1.63(1.07–2.47)	0.022		
Attitude towards extramarital sex	Disagreement	1			
	Agreement	1.77(1.34–2.32)	< 0.001		
Condom use efficacy	Unconfident	1			
	Confident	0.29(0.21–0.39)	< 0.001	0.48(0.34–0.69)	< 0.001
	Very confident	0.12(0.08–0.18)	< 0.001	0.26(0.16–0.43)	< 0.001
Perceived risk of HIV infection	No	1			
	Yes	1.91(1.36–2.68)	< 0.001	2.05(1.33–3.16)	0.001
Early sexual debut	No	1			
	Yes	2.55(1.95–3.33)	< 0.001	1.53(1.07–2.20)	0.021
Condom use at the first intercourse	No	1			
	Yes	0.07(0.05–0.09)	< 0.001	0.08(0.06–0.12)	< 0.001
Multiple sexual partnership	No	1			
	Yes	1.75(1.34–2.28)	< 0.001		

## Discussion

Risky sexual behaviors remained significant public health challenges among young males [2]. We found that of the participants, 27.74% initiated sex before age 18, which was higher than the 23.9% of a national level study [40]. The proportions of reporting multiple sexual partnership and not using condoms at the latest sexual intercourse were 48.60% and 16.52% respectively.

Early sexual debut was a recurring problem with varied negative health outcomes such as increased rate of acquiring STDs and higher possibility of unprotected sexual behaviors [36, 41]. This study, echoing previous results [26, 42], revealed a worrisome trend that male college students aging younger were more likely to initiate sex early. The lowering trend of sexual debut age put young people particularly susceptible to HIV and other STDs, because they might be exposed to more sexual partners subsequently and unprotected sexual behaviors than their counterparts who debuted late [43]. According to the results, male students who had early sexual debut were 3.66 times and 1.53 times more likely to have multiple sexual partners and condomless sex at the latest intercourse, respectively. The study also showed being sexual minorities was positively associated with early sexual debut. Adolescents are reported to be naturally predisposed to escalated urge for sexual activities from puberty. This inclination might be more pronounced among those with sexual identity confusion, because they tried to engage in early sex experimentation to determine sexual orientation [36, 44]. These findings indicated that programs aiming at delaying sexual debut among youth was an important issue of concern.

Multiple sexual partnership was common among youth [22, 45]. A study in Malawi revealed that 69% of young males had more than one sexual partners [46]. Compared to a national meta-analysis report in China [13], the rate of having more than one sexual partner was higher in our results. Ethnic minority male students were more likely to report multiple sexual partnership, which might be explained by different social norms and values concerning sex and marriage [47]. The odds of having multiple sexual partners was higher among male students who accepted extramarital sex. This revealed that sexuality, including ethics of sexual activity and pleasure, should be incorporated into sex education in college.

Condom use was one of the most reliable indicators assessing sexual behavior risks [45]. A typically effective strategy for promoting condom use was increasing condom use efficacy, which was supposed to explain more than 50% of the variance in use intentions and 33% in actual use [48]. We found that condom use efficacy, in terms of insistence, negotiation, rejection, was negatively correlated with condomless sex, and more than half reported unconfident among the participants who did not use condoms. This highlighted health intervention might underscore assertiveness in terms of condom use as well as communication skills with sexual partners [49].

A previous study indicated that no statistical difference of safe sex rate was found among students with different levels of HIV knowledge [19]. However, our study demonstrated that HIV prevention knowledge cast significant impact on condom use behavior. A probable reason of the conflicts in these studies [14, 19, 23], was whether current sex related education could meet the demands of the target population or not [50]. A qualitative research in the USA in 2019 pointed out, received sex education was not helpful and themes of potential improvements were advised to be upon diverse sexual behaviors and identities, and social contexts about sex [51]. Changes to more detailed and customized sex education about HIV prevention knowledge and safe sexual behaviors might be considered.

This study also indicated a positive correlation between heightened HIV risk perceptions and condomless sex at the latest sexual behavior among male college students. Several hypotheses might be accountable. First, the perceived high risk resulted from failure of condom use at the latest sex. Second, despite the awareness of HIV risk infection, young males still chose not to use condoms, which implied that the transition from cognition to adopting safe behaviors was interrupted. A few studies have stressed the threating existence of risk discordance recently [49, 52], the phenomena in our study, however, was even complicated and needed further exploration to identify the influencing factors.

This study had several limitations. First, no causality could be concluded given the cross-sectional study design. Second, participants were enrolled from vocational college schools, as a consequence of which may induce selection bias. Third, this survey was conducted during Covid-19 pandemic. The societal mobility restrictions in response to the pandemic might alter the pattern of students’ behaviors. In addition, when addressing sensitive questions related to sexual behaviors for instance, answers from the participants may be subject to information bias.

## Conclusion

Our study demonstrated the prevalence of early sexual debut, multiple sexual partnership and condomless sex among male college students, as well as the correlations between risky behaviors and HIV knowledge, condom use efficacy and perceived HIV risk. Simultaneously, the trend was noted that aging younger became more susceptible to early sexual debut. As the influence of Covid-19 pandemic decreased, human mobility networks gradually resumed. There was an urgently crucial need for comprehensive sex education curricula by developing customized information on HIV prevention, sexuality, and empowering students with assertiveness and negotiation skills concerning condom use during and before college.

## Data Availability

The datasets analyzed during the current study are available from the corresponding author on reasonable request.

## Abbreviations

HIV/AIDS: Human immunodeficiency virus/acquired immunodeficiency syndrome
PLWH: People living with HIV
STDs: Sexually transmitted diseases
CI: Confidence interval
AOR: Adjusted odds ratio
SD: Standard deviation
